# Substitution of 25% of chemical fertilizer nitrogen with organic amendments nitrogen reduces N_2_O emissions from tea plantation soils in subtropical China

**DOI:** 10.3389/fmicb.2025.1715814

**Published:** 2025-11-27

**Authors:** Qiao Sun, Yu Cheng, Suzhen Yin, Xiaojing Sun, Xiaoyue Song, Xinyue Song, Wanyu Shen, Biao Meng, Xinxiao Wang, Xintong Lv, Hui Li, Haiyang Yu

**Affiliations:** 1 College of Resources and Environment, Anhui Agricultural University, Hefei, China; 2 Shandong Provincial Research Institute of Coal Geology Planning and Exploration, Jinan, China; 3 Soil and Fertilizer Institute, Anhui Academy of Agricultural Sciences (National Agricultural Experimental Station for Soil Quality, Taihe)/Anhui Provincial Key Laboratory of Nutrient Cycling and Arable Land Conservation, Hefei, China

**Keywords:** tea plantation, emission factor, nitrous oxide, organic fertilizer, denitrification genes

## Abstract

Tea plantation soils are recognized hotspots for nitrous oxide (N_2_O) emissions due to excessive amounts of nitrogen fertilizer applied, yet the potential of partially substituting chemical fertilizers with organic amendments to mitigate this effect remains insufficiently assessed. This study investigated the impact of partial organic substitution on N_2_O emissions and their underlying mechanisms in a tea plantation soil from southern Anhui, China, by using a 28-day incubation study. Soil samples were subjected to fertilization treatments featuring 25 and 50% substitutions of chemical nitrogen (N) with either pig manure or rice straw. We measured N_2_O emissions alongside key soil parameters, including soil pH, microbial biomass carbon (MBC), ammonium N (NH_4_^+^-N) content, nitrate N (NO_3_^−^-N) content, and the abundances of *nirS*, *nirK*, and *nosZ* genes. The results showed that while N application elevated N_2_O emissions, partial substitution with organic fertilizers effectively mitigated them. Among all treatments, a 25% substitution ratio was optimal, with straw return yielding superior reduction effects compared to pig manure. Structural equation modelling (SEM) revealed that, compared to conventional fertilization, partial replacement of chemical fertilizers with organic amendments elevated soil pH and MBC content, which subsequently mediated the abundances of *nirS*, *nirK*, and *nosZ* genes via the modulation of NH_4_^+^-N and NO_3_^−^-N content, ultimately leading to reduced N_2_O emissions. Collectively, these findings indicate that a lower substitution ratio of organic fertilizer can effectively reduce N_2_O emissions from tea plantation soils, thereby providing a scientific basis for achieving “carbon neutrality” and promoting the sustainable development of tea agroecosystems.

## 1 Introduction

Among greenhouse gases (GHGs), nitrous oxide (N_2_O) is the third most significant contributor to global radiative forcing, after carbon dioxide (CO_2_) and methane (CH_4_) (Thompson et al., 2019; Zhu et al., 2025). Since the Industrial Revolution, atmospheric N_2_O concentrations have risen markedly from approximately 270 ppbv to 339 ppbv (Lan et al., 2025). Meanwhile, the growth rate has accelerated progressively, reaching 1.15 ± 0.12 ppbv yr.^−1^ in 2021–2024, highlighting the urgency of implementing effective mitigation strategies (Lan et al., 2025). Critically, over a 100-year period, the global warming potential of a single molecule of N_2_O is 273 times that of CO_2_ (IPCC, 2021), emphasizing the need for heightened global attention to N_2_O emissions and that action to reduce them must not be delayed.

Agricultural soils constitute a major anthropogenic source of N_2_O emissions (2.2–4.8 Tg N yr.^−1^) (Li et al., 2024; Xu et al., 2020; Zhu et al., 2025), among which tea (*Camellia sinensis* L.) plantations are identified as a notable hotspot (Akiyama et al., 2023; Wang et al., 2022; Yu et al., 2023). Annual N_2_O emissions from tea plantation soils were estimated to be around 44.5 Gg N yr.^−1^, contributing approximately 1.5–12.7% of total anthropogenic N_2_O emissions (Yu et al., 2023). In actual agricultural management, in pursuit of higher tea yields, farmers often apply excessive amounts of synthetic fertilizers, which not only degrade soil quality (e.g., leading to acidification and nutrient depletion) but also further stimulate substantial N_2_O emissions, thereby creating a positive feedback loop for climate warming (Huang et al., 2025a; Wang et al., 2020). Empirical studies have demonstrated a strong linear relationship between nitrogen (N) fertilizer application rates and N_2_O emissions in tea plantations (Han et al., 2021; Yu et al., 2023), with autotrophic nitrification processes accounting for 50–57% of total N_2_O production (Cheng et al., 2015). However, previous studies also demonstrated that denitrification can contribute nearly 75% of total N_2_O emissions in tea plantation soils (Chen et al., 2017; Jumadi et al., 2008). These findings indicate that there is currently no consensus on the main pathways of N_2_O emission in tea plantation soils (Tu et al., 2025). This uncertainty may be attributed to differences in tea plantation cultivation areas, various planting habits, and diverse management practices (Yu et al., 2023), which result in variations in N cycling genes abundance, soil properties, microbial diversity, and community structure.

Numerous factors affect the production and emission of N_2_O in tea plantation soils. These include climatic conditions such as temperature and rainfall, soil properties like soil pH, ammonium N (NH_4_^+^-N) and nitrate N (NO_3_^−^-N) content, microbial community composition, as well as management practices including green manure intercropping and optimized fertilization (Yu et al., 2023). In the staple crop systems, partial substitution of chemical fertilizers with organic alternatives has proven effective in increasing crop aboveground N uptake by 7% and N fertilizer utilization rate by 10% and reducing N_2_O emissions by 3–12% (Xia et al., 2017a). This mitigating effect of organic fertilizers on N_2_O emissions can be attributed not only to enhanced crop N uptake and the slower nutrient release rate from these fertilizers, but also to a substitution effect (Chivenge et al., 2011; Wang et al., 2015). This effect strengthens the capacity of soil microorganisms to immobilize inorganic N, thereby reducing soil available N content (Xia et al., 2017a). As a result, the substrate supply necessary for nitrification and denitrification processes is limited, further suppressing N_2_O production. However, in tea plantations, the impact and underlying mechanisms of such substitutions remain poorly understood.

In this study, we hypothesized that partially substituting chemical fertilizers with organic amendments (rice straw and pig manure) would directly or indirectly modulate N_2_O emissions by altering the abundance of key functional genes involved in N cycling. Furthermore, the substitution ratio was expected to be a critical factor determining the outcome. To test these hypotheses, a microcosm experiment was conducted with the following objectives: (i) to examine the effects of replacing 25% and 50 of chemical fertilizers with organic fertilizers on N_2_O emissions in tea plantation soils, and (ii) to elucidate the underlying microbial mechanisms driving changes in N_2_O production and emission. The results are expected to offer important insights for the sustainable management of tea plantations and contribute to efforts toward agricultural carbon neutrality.

## 2 Materials and methods

### 2.1 Soil sampling

Soil samples were collected in December 2023 from a typical tea plantation located in Xiuning County, southern Anhui Province, China (29°55′ 12′′N, 118°9′ 31′′ E). The region experiences a subtropical monsoon climate and is renowned for its green tea production. The mean annual precipitation and temperature are 1937 mm and 16.3 °C, respectively. This tea plantation has been established for over 60 years and receives fertilization twice annually: a base fertilizer consisting of both organic and inorganic inputs, and a top-dressing of inorganic fertilizers (urea), totaling approximately 200 kg N hm^−2^ yr.^−1^.

Soil sampling followed the protocols outlined in the *Soil Agro-Chemical Analyses* (Lu, 2000). A complete randomized block design was employed, with three replicated plots randomly distributed across the plantation. From each plot, five soil cores were collected from the surface layer (0–20 cm) using a soil auger according to the five-point sampling method. All cores were then combined to form a single composite sample. Visible plant debris and small stones were removed from the composite samples. The samples were immediately stored in a portable cooler at 4 °C for transport to the laboratory. Upon arrival, the soil was sieved (≤2 mm) and stored at 4 °C until analysis.

### 2.2 Incubation experiment

Soils were incubated following the methodology of Han et al. (2025). The experimental treatments were as follows: (1) Control (CK), no N fertilizer amendent; (2) conventional fertilization (CN), amendment with urea only; (3) 25% N substitution with pig manure (25%PN), 25% N derived from pig manure and 75% from urea; (4) 25% N substitution with rice straw (25%RN), 25% N derived from rice straw and 75% from urea; (5) 50% N substitution with pig manure (50%PN), 50% N derived from pig manure and 50% from urea; (6) 50% N substitution with rice straw (50%RN), 50% N derived from rice straw and 50% from urea (Supplementary Figure S1).

Prior to the incubation experiment, fresh soil samples (10 dry weight) were placed into 120 mL serum bottles and pre-incubated at 25 °C for 1 week to stabilize microbial activity. After pre-incubation, the respective amendments—urea, pig manure (granular form after fermentation and maturation, ground into powder before application), and rice straw (milled into powder)—were added to the brown serum bottles according to treatment specifications and thoroughly mixed with the soil. Detailed application rates are provided in Supplementary Table S1. Sterile water was then added to adjust the soil moisture content to 60% of the maximum field water holding capacity (WHC). Each treatment was replicated 15 times. All bottles were sealed with rubber stoppers and incubated in the dark at 25 °C for 28 days. Throughout the incubation period, the bottles were weighed every 2 days to check moisture loss, and deionized water was added as needed to maintain constant soil moisture.

### 2.3 Gas and soil sampling and analysis

Gas sampling was conducted on days 1, 7, 14, 21, and 28 of the incubation (Supplementary Figure S1). Prior to each sampling event, the bottles were uncrapped and vented in a fume hood for 30 min to refresh the headspace and equilibrate with atmospheric pressure (Zheng et al., 2020). After 24 h, a 1 mL gas sample was collected from the headspace of each brown serum bottle using a 10 mL gas-tight syringe. Each sample was immediately diluted to 20 mL with pure N_2_ (Shen et al., 2024). The concentrations of N_2_O in the diluted samples were analyzed using a gas chromatograph (Agilent 7890B, Agilent, USA) equipped with an electron capture detector (ECD). The calculation of N_2_O emission rates and cumulative N_2_O emissions was detailed in Supplementary File 1.

Destructive soil sampling was performed concurrently with gas sampling on the same days. For each sampling time point, three replicate bottles per treatment were destructively sampled. The soil from each bottle was divided into two portions. One portion was air-dried for the analysis of soil physicochemical properties, while the other portion was stored at −80 °C for subsequent DNA extraction and quantification of microbial functional genes.

Soil moisture content was determined gravimetrically by drying the soil at 105 °C for over 10 h until a constant weight was achieved. Soil pH was measured in a 1:2.5 (w/v) soil-to-water slurry using a pH meter (Mettler-Toledo, Switzerland). The concentration of ammonium N (NH_4_^+^-N) and nitrate N (NO_3_^−^-N) was extracted with 2 M KCl (1:10 soil-to-solution ratio, w/v), filtered through a 0.45 μm membrane, and analyzed using a continuous flow colorimeter (SEAL Auto Analyzer 3, UK). Microbial biomass carbon (MBC) was determined by the chloroform fumigation-potassium sulfate leaching method, and the value was calculated as the difference in extractable organic carbon between fumigated and non-fumigated soil divided by a conversion factor of 0.45. Detailed methodologies for these analyses are provided in Text S2.

### 2.4 DNA extraction and quantitative PCR

Total genomic DNA was extracted from 0.5 g of the frozen dry soil (previously stored at −80 °C) using the DNeasy^®^PowerSoil^®^Pro Kit (QIAGEN, Germany) according to the manufacturer’s protocol (Zheng et al., 2019). The abundances of the key functional genes associated with N cycling (*nirS*, *nirK*, and *nosZ* gene copies) were quantified by quantitative real-time PCR on a LightCycler Roche 480 (Roche Molecular Systems, Switzerland), following the methodology described by Throback et al. (2004). The sequences of gene-specific primers and the detailed qPCR protocols were listed in Supplementary Table S2. Standard curves were constructed by subjecting tenfold serial dilutions of the plasmids to qPCR under the same conditions used for the soil DNA samples. For each target gene, plasmids were constructed by cloning PCR-amplified fragments into vectors, transforming them into *Escherichia coli*, and purifying them with a plasmid extraction kit. Plasmid concentrations were determined using a NanoDrop spectrophotometer (Thermo Scientific, USA), and copy numbers were calculated based on molecular weight and fragment size. All qPCR reactions, including no-template controls and serial standard curve samples, were performed in triplicate. The resulting amplification efficiencies ranged from 86 to 98%, with all standard curves exhibiting strong linearity (*R^2^* = 0.999).

### 2.5 Statistical analysis

All statistical analyses were performed using SPSS 25.0 (IBM SPSS, USA). Differences in soil chemical and biological characteristics among treatments were evaluated by one-way analysis of variance (ANOVA), followed by Duncan’s multiple range test for *post-hoc* comparisons; a probability value of *p* < 0.05 was considered statistically significant. The relationships between N_2_O emissions and various abiotic and biotic factors were examined using linear and polynomial regression analyses. Structural equation modelling (SEM) was performed using IBM SPSS Amos 19 (Amos Development Corporation, USA) to elucidate the causal pathways through which the partial substitution of chemical fertilizers with organic amendments (all the partial substitution treatments) influences N_2_O emissions. The goodness-of-fit for SEM was assessed by the chi-square (*χ^2^*) test and *p*-value, goodness-of-fit index (GFI), and root mean square error of approximation (RMSEA). All figures were generated using OriginPro 2024 (OriginLab, USA) or PowerPoint (Microsoft, USA).

## 3 Results

### 3.1 N_2_O emissions

**Figure 1:**
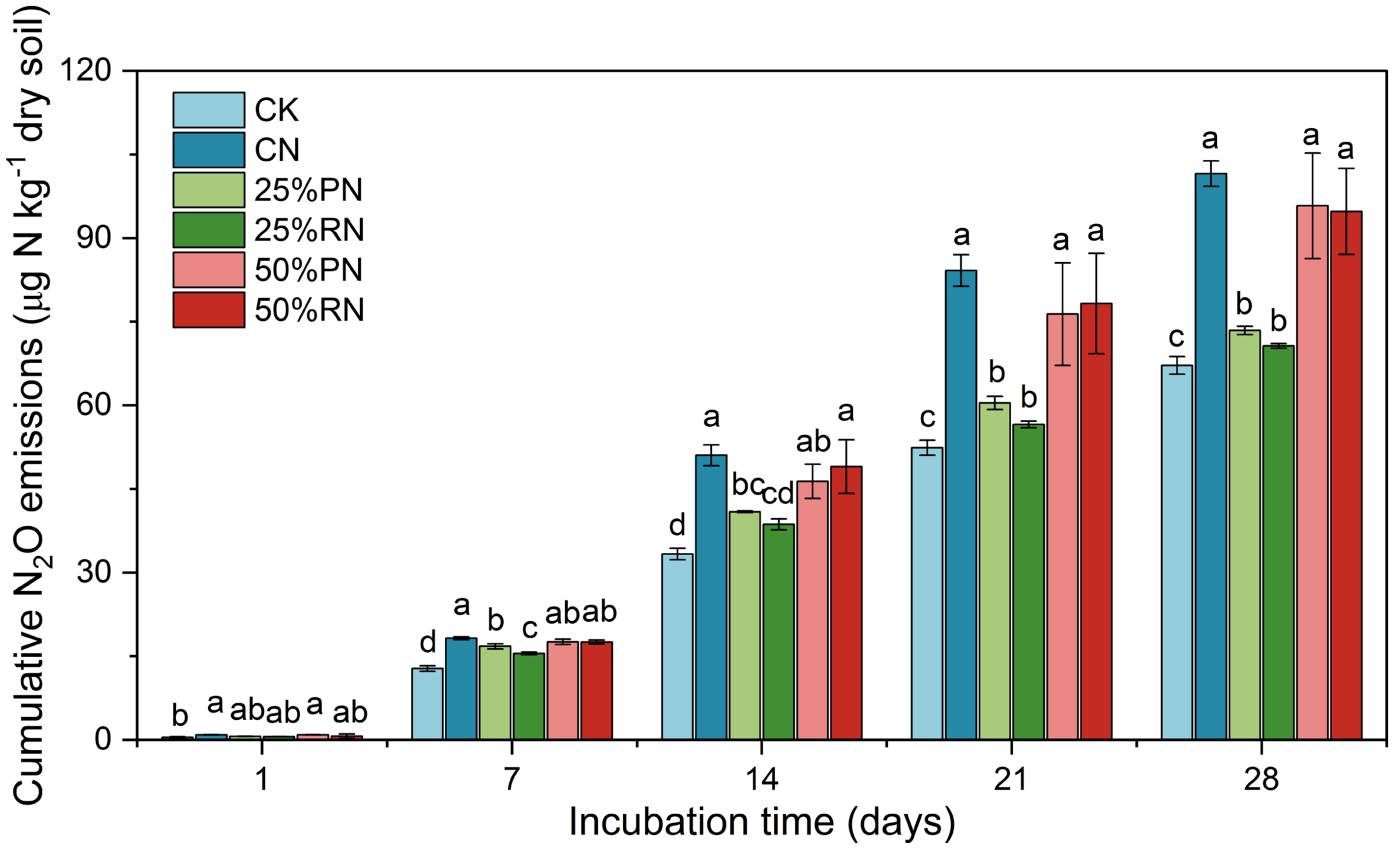
Cumulative N_2_O emissions in tea plantation soils during incubation time. CK, no N fertilizer amendent; CN, amendment with urea only; 25%PN, 25% N derived from pig manure and 75% from urea; 25%RN, 25% N derived from rice straw and 75% from urea; 50%PN, 50% N derived from pig manure and 50% from urea; 50%RN, 50% N derived from rice straw and 50% from urea. Values represent mean ± SE (*n* = 3). Different lowercase letters indicate significant differences among treatments at *p* < 0.05.

During the entire incubation period, cumulative N_2_O emissions increased in all treatments (Figure 1, Supplementary Figure S2). Relative to the CK treatment, all other treatments exhibited an increase in cumulative N_2_O emissions after 7 days (*p* < 0.05). In contrast, partial substitution of chemical fertilizer with organic amendments reduced cumulative N_2_O emissions compared to the CN treatment. Specifically, the 25%PN and 25%RN treatments resulted in reductions of 28 and 30%, respectively (*p* < 0.05), whereas the reductions observed in the 50%PN (6%) and 50%RN (7%) treatments were not statistically significant (*p* > 0.05).

### 3.2 Soil physicochemical properties

**Figure 2:**
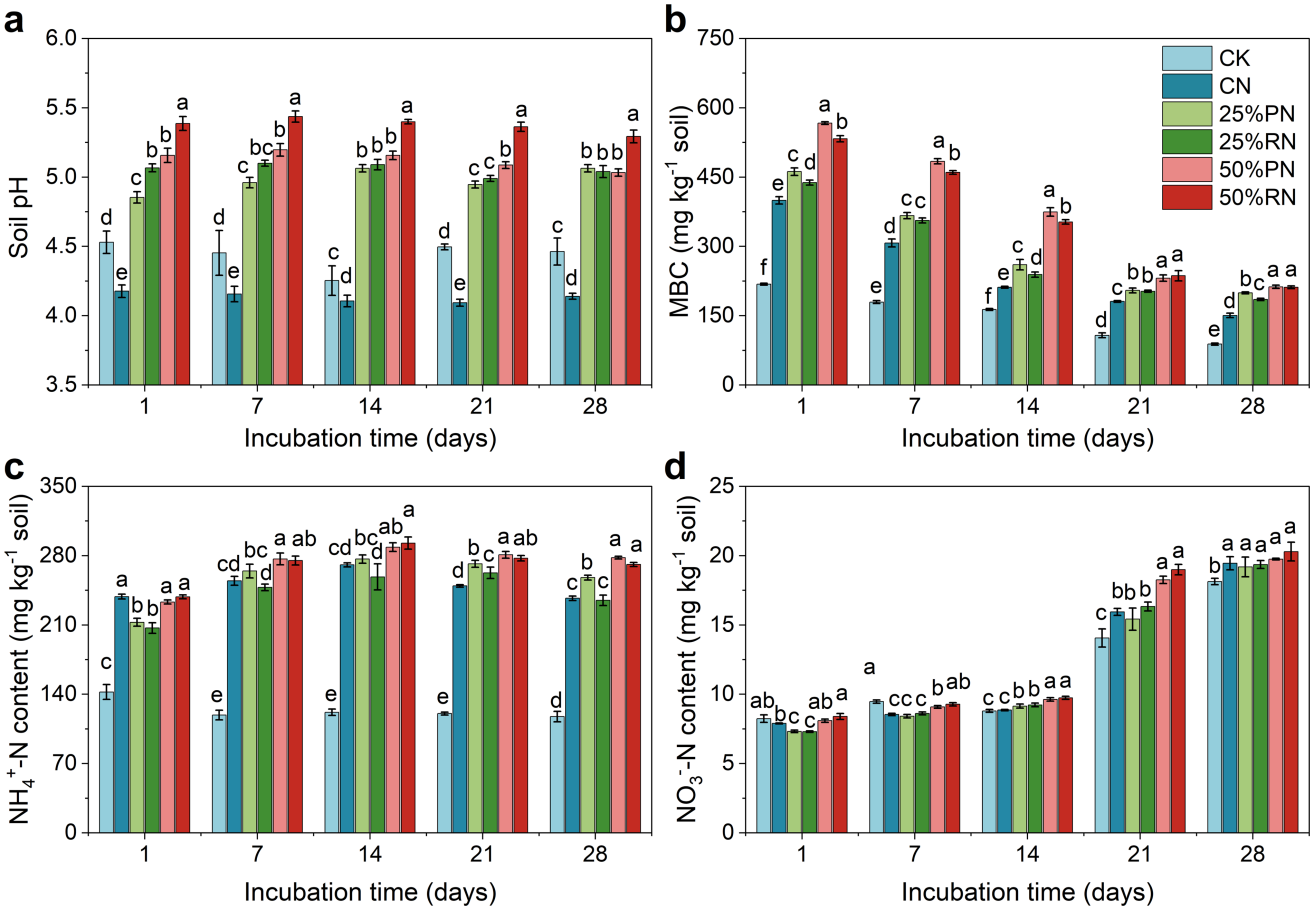
Dynamics of soil pH **(a)**, microbial biomass carbon (MBC) **(b)**, ammonium content (NH_4_^+^-N) **(c)**, and nitrate content (NO_3_^−^-N) **(d)** in tea plantation soils during incubation time. CK, no N fertilizer amendent; CN, amendment with urea only; 25%PN, 25% N derived from pig manure and 75% from urea; 25%RN, 25% N derived from rice straw and 75% from urea; 50%PN, 50% N derived from pig manure and 50% from urea; 50%RN, 50% N derived from rice straw and 50% from urea. Values represent mean (*n* = 3). Different lowercase letters indicate significant differences among treatments at *p* < 0.05.

The dynamics of soil pH, MBC, NH_4_^+^-N, and NO_3_^−^-N throughout the incubation period are presented in Figure 2. Soil pH remained relatively stable and changed minimally across all treatments (Figure 2a). However, compared to the CK treatment, soil pH decreased in the CN treatment (*p* < 0.05), whereas it increased in all treatments with partial organic fertilizer substitutions (*p* < 0.05), with the 50% RN treatment exhibiting the most pronounced increase. Soil MBC showed a general declining trend over time in all treatments (Figure 2b). Exogenous N application increased soil MBC (*p* < 0.05), and MBC content in the organic substitution treatments was higher than in the CN treatment, with the 50% PN treatment showing the greatest effect (*p* < 0.05).

Compared to the CK treatment, exogenous N application increased NH_4_^+^-N content in CN and all organic substitution treatments (*p* < 0.05), with values remaining stable over time (Figure 2c). After 7 days, NH_4_^+^-N contents were higher in the 50%PN and 50%RN treatments than in the CN treatment (*p* < 0.05), despite no difference between the CN treatment and the 25% organic substitution treatments. Additionally, NO_3_^−^-N content of all treatments showed no change in the initial 14 days but increased by the end of the incubation (Figure 2d).

### 3.3 Abundances of *nirS*, *nirK*, and *nosZ* genes

**Figure 3:**
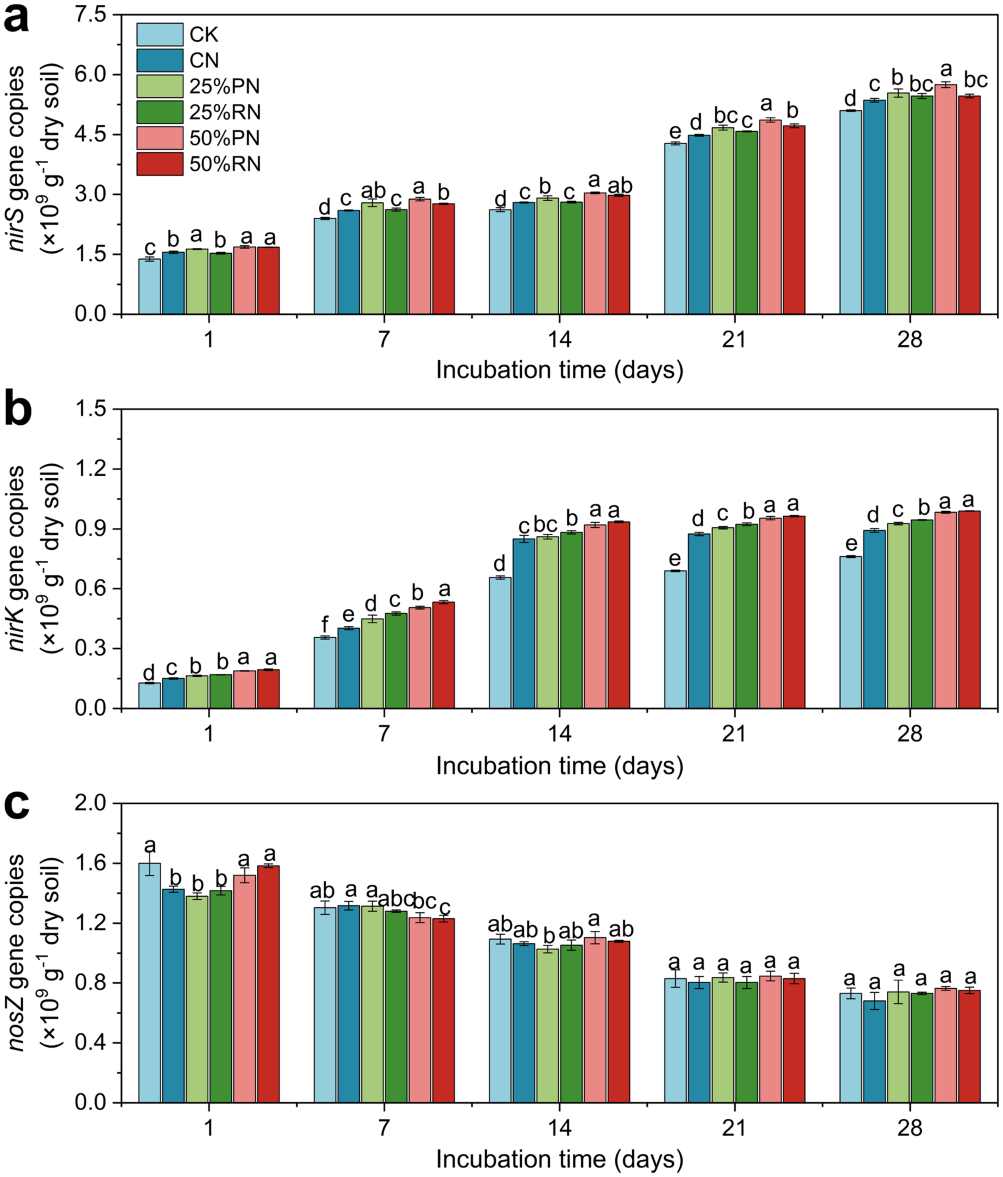
The copy numbers per g dry soil of the N cycle functional genes *nirS* **(a)**, *nirK* **(b)**, and *nosZ* **(c)** in tea plantation soils during incubation time. CK, no N fertilizer amendent; CN, amendment with urea only; 25%PN, 25% N derived from pig manure and 75% from urea; 25%RN, 25% N derived from rice straw and 75% from urea; 50%PN, 50% N derived from pig manure and 50% from urea; 50%RN, 50% N derived from rice straw and 50% from urea. Values represent mean (*n* = 3). Different lowercase letters indicate significant differences among treatments at *p* < 0.05.

The abundance of the key functional genes that are key during the corresponding processes exhibited considerable temporal variation throughout the incubation period (Figure 3). The abundances of both *nirS* and *nirK* genes increased over time in all treatments, whereas the abundance of *nosZ* gene gradually decreased. Compared to the CK treatment, exogenous N application increased the abundances of *nirS* and *nirK* genes (*p* < 0.05, Figures 3a,b). Furthermore, all organic substitution treatments supported higher abundances of these two genes than the CN treatment (*p* < 0.05).

For *nosZ* gene, its abundance in the 50% RN and 50% PN treatments was comparable to that in the CK treatment during the early incubation stage (Figure 3c). In contrast, the abundance of the *nosZ* gene in the 25% PN and 25% RN treatments was similar to the CN treatment and lower than in the CK treatment (*p* < 0.05). After 7 days of incubation, the *nosZ* gene abundance became statistically similar across all treatments.

### 3.4 Relationship between N_2_O emissions and soil physicochemical properties, as well as abundances of *nirS*, *nirK*, and *nosZ* genes

**Figure 4:**
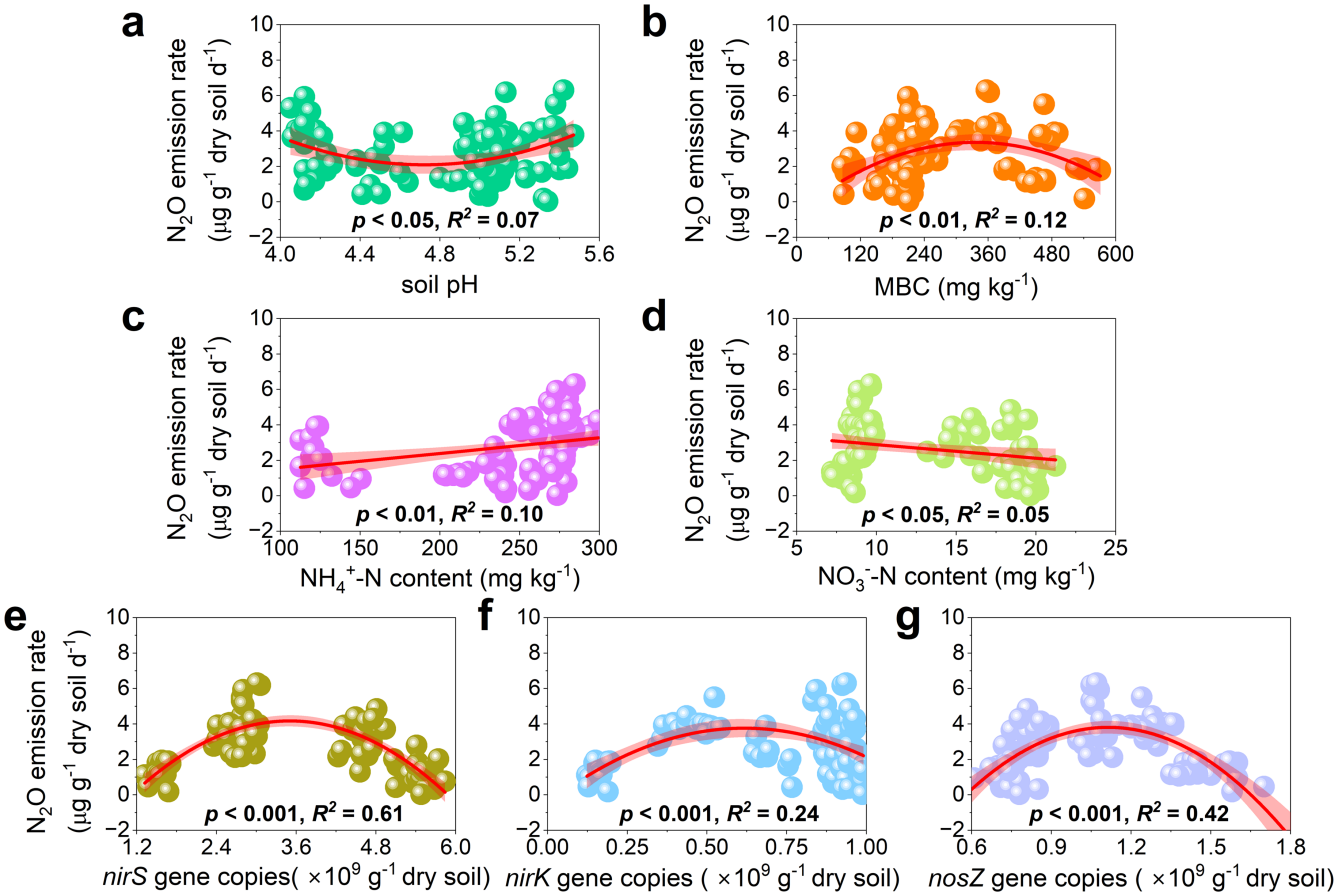
Relationships between N_2_O emission rates with soil pH **(a)**, MBC **(b)**, NH_4_^+^-N content **(c)**, NO_3_^−^-N content **(d)**, *nirS* gene copies **(e)**, *nirK* gene copies **(f)**, and *nosZ* gene copies **(g)** (*n =* 90).

Regression analysis revealed distinct relationships between N_2_O emission rates and various soil physicochemical properties, as well as abundances of *nirS*, *nirK*, and *nosZ* genes (Figure 4). A significant upward-opening parabolic relationship was observed with soil pH (*p* < 0.05, Figure 4a), whereas significant downward-opening parabolic relationships were found with MBC and the abundances of *nirS*, *nirK*, and *norZ* genes (*p* < 0.01, Figures 4b,e–g). Furthermore, N_2_O emission rates showed a positive linear correlation with NH_4_^+^-N content and a negative linear correlation with NO_3_^−^-N content (*p* < 0.05, Figures 4c,d). Notably, an initial Pearson correlation analysis did not reveal significant linear correlations between gene abundances and N_2_O emission rates (Supplementary Figure S3).

**Figure 5:**
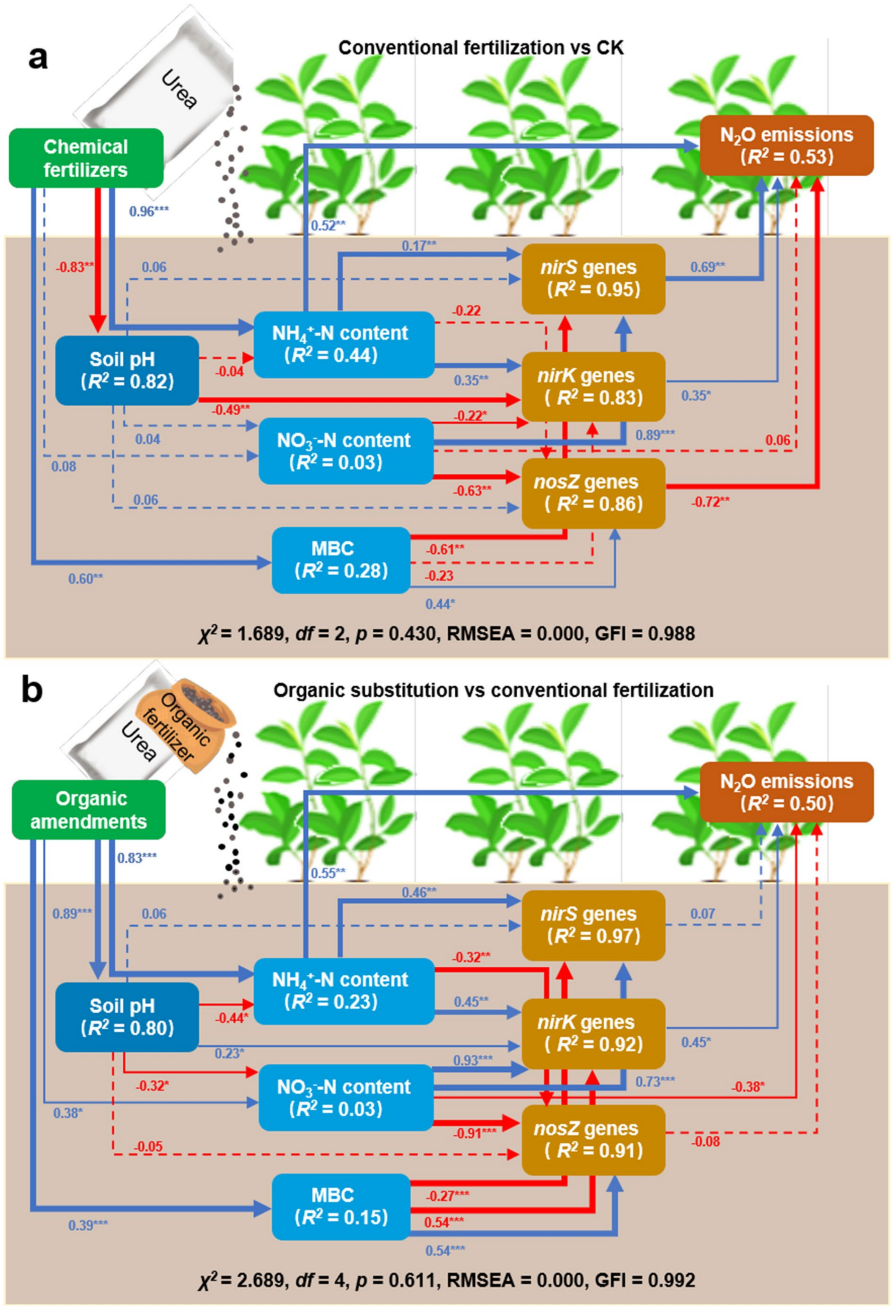
Structural equation modelling (SEM) illustrating the direct and indirect effects of conventional fertilization **(a)** and the partial substitution of chemical fertilizers with organic amendments **(b)** on N_2_O emissions, respectively. The blue arrows indicate significant positive relationships, whereas the red arrows indicate significant negative relationships, where the significance level was set at *p < 0.05; **p < 0.01; ***p < 0.001, respectively. Numbers beside the arrows are standardized coefficients. The *R*^2^ values indicate the degree of the variable interpreted by all paths from the combination of the fixed and random effects.

SEM revealed that, compared to CK treatment, conventional fertilizer application increased NH_4_^+^-N content, thereby promoting N_2_O emissions. It also reduced soil pH, regulated MBC content, enhanced *nirS* gene abundance, and consequently stimulated N_2_O emissions (Figure 5a). Under this fertilization regime, these interacting factors collectively explained for 53% of the variation in N_2_O emissions. In contrast to conventional fertilization, organic amendments partially replaced chemical fertilizers elevated soil pH and MBC content, thereby suppressing the abundances of the key functional genes (*nirS*, *nirK*, and *nosZ*) involved in N cycling through the modulation of NH_4_^+^-N and NO_3_^−^-N content, which led to reduced N_2_O emissions (Figure 5b). Collectively, the controlling factors in combination explained for the 50% variation of N_2_O emissions.

## 4 Discussion

### 4.1 Effects of the partial substitution of chemical fertilizers with organic amendments on N_2_O emissions in tea plantations

Due to the rising demand for tea, the application of N fertilizers in Chinese tea plantations must be intensified to ensure yield stability and enhance economic returns (Bharadwaj et al., 2025; Yu et al., 2023). However, such practice inevitably elevates N_2_O emissions from tea plantation soils (He et al., 2019; Wang et al., 2020). Our results confirmed that the sole application of chemical N fertilizers significantly promoted N_2_O emissions from tea plantation soil (Han et al., 2025; Wang et al., 2022; Wang et al., 2020; Yu et al., 2023). This was primarily driven by an increase in NH_4_^+^-N content, which stimulated nitrification, coupled with a rise in NO_3_^−^-N content that enhanced denitrification—both processes contributing to higher N_2_O emissions (Toteva et al., 2024). Notably, chemical N fertilization further acidified tea plantation soil (Figures 4, 5), which may partially suppress denitrification activity. Although the transcription of the *nosZ* gene may remain unaffected under low pH, the translation, protein assembly, or enzymatic activity of *nosZ* are likely impaired (Liu et al., 2010; Tu et al., 2025). Consequently, in tea plantation soils subjected to long-term chemical fertilization, N_2_O emissions may be progressively enhanced (Huang et al., 2025b). Additionally, both our findings and previous studies directly or indirectly identify soil pH as a key regulator of microbial N cycling (Qiu et al., 2024; Wang et al., 2022). Therefore, effective mitigation of N_2_O emissions from tea plantations should focus on management strategies that actively regulate soil pH.

Organic management has been established as a viable approach for the sustainable development of tea plantations, primarily by mitigating soil acidification and enhancing soil N transformation capacity (Jiang et al., 2025; Wu et al., 2025). This study compared the effects of N fertilizer application and its partial replacement with organic fertilizer at varying substitution rates on N_2_O emissions and associated environmental factors in tea plantation soils (Figures 2, 3). The results demonstrated that partial substitution of chemical fertilizer with organic amendment significantly reduced N_2_O emissions, although the reduction efficiency did not exhibit a linear response to the substitution rate, which was consistent with our hypothesis. Among the tested ratios, the 25% replacement level was found to be the most effective in mitigating N_2_O emissions (Figure 1).

### 4.2 Mechanisms of N_2_O emission reduction in tea plantation soils following partial substitution of chemical fertilizers with organic amendments

Partial substitution of chemical fertilizers with organic amendments represents an effective and environmentally friendly strategy for N_2_O mitigation in tea plantations, achieved through integrated improvement of the soil environment. The underlying mechanism for N_2_O reduction following organic amendment involves a synergistic interplay of physical, chemical, and biological processes rather than a single pathway (Tu et al., 2025). Specifically, organic fertilizer improves soil structure, thereby minimizing the formation of strongly anaerobic microsites conducive to N_2_O production (Han et al., 2022). It also elevates soil pH, which enhances the activity of N_2_O reductase (the enzyme responsible for the reduction of N_2_O to N_2_) (Bharadwaj et al., 2025). Furthermore, the incorporation of organic materials supplies a sustained source of organic carbon, facilitating complete denitrification and reducing N_2_O accumulation (Tu et al., 2025). The gradual N release from organic sources also lowers the substrate availability for nitrification and denitrification, thereby limiting N_2_O generation at its origin (Wang et al., 2015). Additionally, organic fertilization helps shape a microbial community enriched with N_2_O-reducing organisms, further reinforcing the sink capacity for N_2_O (Rose et al., 2020). However, in the present study, although organic substitution increased the abundances of the denitrification genes *nirS* and *nirK*, the concurrent rise in soil pH decreased substrate concentration, which likely contributed to the observed reduction in N_2_O emissions. Conversely, the abundance of the key N_2_O-consuming gene *nosZ* remained unchanged, which may explain why no significant enhancement in N_2_O reduction was detected.

In this study, a parabolic relationship was observed between soil pH and N_2_O emission rate (Figure 4a), suggesting the potential existence of a critical pH threshold. N_2_O emissions increased when soil pH fell either below or above this threshold. Variations in the proportion of organic substitution induced changes in soil pH, which in turn influenced the abundance of key genes involved in the N cycle (*nirS*, *nirK*, and *nosZ*), thereby modulating N_2_O emissions in tea plantation soils. These findings underscore the essential role of the reductases encoded by these denitrification genes in the N_2_O production process (Xu et al., 2018).

MBC can serve as an indicator of soil N supply capacity (Han et al., 2025). Compared with conventional chemical fertilizer treatment, a moderate proportion of organic substitution increased soil MBC in tea plantation soils. The rise in MBC likely activated key enzymes and facilitated N_2_O consumption (Figure 5b). However, when a high proportion of organic fertilizer was applied, the further accumulation of MBC (driven by excessive organic matter) appeared to suppress the activity of these key enzymes, disrupting the balance between N_2_O production and consumption and ultimately leading to enhanced N_2_O accumulation (Han et al., 2025). In this study, the effects of pig manure organic fertilizer and rice straw organic fertilizer were not significantly different. The reason might be that the cultivation period was relatively short. There are certain differences in the impact of long-term straw return and pig manure return on N_2_O emissions from food crops (Chen et al., 2024a). This might be because the efficiency of N supply by pig manure and straw is different. Generally speaking, the N release capacity of straw is lower than that of pig manure (Dong et al., 2018). This is why in our experiment, the 25% straw substitution with chemical fertilizers achieved the best results.

### 4.3 Implications and limitations

This study demonstrated that a 25% substitution of chemical fertilizers with organic amendments (particularly rice straw) represents a promising strategy for mitigating N_2_O emissions in acidic tea plantation soils. These findings provide a scientific basis for refining fertilization practices in subtropical tea plantations and contribute to the development of low-emission agriculture, supporting China’s “carbon neutrality” goals. From a practical perspective, the 25% substitution rate balances environmental benefits with agricultural feasibility, making it a readily adoptable practice for local tea farmers.

However, several limitations should be considered when interpreting these results. First, this study was conducted under controlled laboratory conditions, which may not fully capture the complex interactions present in field environments, such as fluctuating temperature, precipitation, and root effects. Temperature, in particular, has been identified as a critical factor influencing N_2_O emissions (Poh et al., 2015). It is also worth noting that tea plantations in China are often situated at higher altitudes (where fertilization is commonly applied in winter), conditions that may further suppress N_2_O emissions. Second, the relatively short incubation period might not reflect the long-term dynamics of soil carbon and N turnover, or their subsequent effects on N_2_O emissions. With the widespread adoption of organic substitution practices in China’s “Chemical Fertilizer Reduction Action,” the resulting shifts in field management are expected to significantly alter N_2_O emissions from tea plantations and farmland ecosystems (Xia et al., 2017b). Third, while the abundances of key genes (*nirS*, *nirK*, *nosZ*) were quantified, the composition and transcriptional activity of the microbial community were not analyzed, leaving the underlying regulatory mechanisms at the microbial functional level incompletely elucidated. Previous studies suggest that organic amendments may influence N_2_O emissions primarily by altering the composition of key microbial communities involved in denitrification, rather than merely changing their gene abundance (Chen et al., 2024b). Future research should therefore incorporate field validation experiments and employ multi-omics approaches to unravel the full microbial functional network governing N_2_O emissions in tea plantations under organic substitution practices. Finally, replacing chemical fertilizers with organic fertilizers is beneficial for the sustainable production of tea plantations. This practice helps maintain soil health, sustain tea yield and quality, and promotes the synergistic improvement of both economic and environmental benefits (Ji et al., 2026). However, further study is still needed to elucidate how partial substitution of chemical fertilizers with organic amendments enhances tea yield and quality through the mediation of soil element cycling and associated changes in microbial community structure and function.

## 5 Conclusion

Our findings demonstrated that partial substitution of chemical fertilizers with organic amendments significantly mitigated N_2_O emissions in strongly acidic tea plantation soils, with the most effective reduction achieved at a 25% substitution ratio. This result confirmed our initial hypothesis. The underlying mechanism primarily involved the elevation of soil pH and the modulation of key microbial functional genes involved in the denitrification pathway. Furthermore, the increase in MBC following organic amendment indirectly influenced N_2_O emissions by shaping the abundance of these denitrification-related functional genes.

## Data Availability

The original contributions presented in the study are included in the article/Supplementary material, further inquiries can be directed to the corresponding authors.
