# Supplementary material for: Substitution of 25% of chemical fertilizer nitrogen with organic amendments nitrogen reduces N2O emissions from tea plantation soils in subtropical China
Source: Front Microbiol. 2025 Nov 27;16:1715814. doi: 10.3389/fmicb.2025.1715814 (PMC12696188; doi:10.3389/fmicb.2025.1715814)
Supplement: Supplementary file 1 [file Data_Sheet_1.docx]

***Supporting information***

**Text S1** N_2_O emission rate and the cumulative N_2_O emission

N_2_O emissions are calculated each day as follows:

F=C_N2O_ × T × ρ × V ÷ M

where, F is the daily emission of N_2_O (μg N kg^-1^ d^-1^); C_N2O_ is the concentration of N_2_O in the headspace of each brown serum bottle (ppbv); T is the time from the previous day’s aeration time to the next day’s uptake measurement time (24 h); 1.25 is the density of N_2_O (1.25 g L^-1^); V is the headspace volume of each brown serum bottle (L); M is the soil mass of each treatment (10 g).

Cumulative N_2_O emissions were calculated using the following equation:

E_N2O_=$\sum_{i=1}^{n}$(F_i_ + F_i+1_) /2 × (t_i+1_ - t_i_)

where E_N2O_ denotes cumulative N_2_O emissions (μg N kg^-1^), F is the N_2_O emission flux (μg N kg^-1^ d^-1^), i denotes gas sampling on day i, t_i+1_ - t_i_ denotes the time between two adjacent measurements (d), and n is the number of measurements.

**Text S2** Determination of soil physicochemical properties

Tea garden soil moisture content was determined using the gravimetric method by weighing 2 g of soil, wrapping it in tin foil, and placing it in an oven at 105°C for at least 10 hours to ensure that the water evaporates completely, and then weighing and recording it to calculate the tea garden soil moisture content.

Soil pH of the tea garden was determined using a pH meter (Mettler-Toledo, Switzerland). 2 g of fresh soil was weighed according to the ratio of water to soil 1:2.5, and the dry soil weight was calculated from the water content, and the corresponding proportion of distilled water was added, and after 5 min of vibration on the oscillator, the meter was centrifuged at 2000 rpm for 3 min. After calibrating the pH meter with buffer according to the pH meter instructions, the probe was placed in the supernatant, and the readings were stabilized to record the pH of the soil samples from the tea gardens.

MBC was determined using the chloroform fumigation-extraction method. Briefly, 5 g soil was weighed into a 25 mL beaker, with three replicates prepared for both fumigated and non-fumigated treatments. All beakers were placed in a vacuum desiccator containing 3-5 pieces of moistened filter paper at the bottom. Also placed inside the desiccator weree a beaker containing 25mL of 1M sodium hydroxide (NaOH) solution and anther beaker holding zeolite soaked with 25mL of choroform. The desiccator was sealed and evacuated using a vacuum pump until the chloroform boiled continuously for 2 minutes. The valve was then closed, and the desiccator was kept in the dark for 24 h. After fumigation, the valve was opened to equalize the pressure. The beakers containing NaOH and chloroform were removed, and residual chloroform was eliminated from the soil by repeated evacuation until no chloroform odor was detected. The non-fumigated control samples were similarly incubated in the dark for 24 h. Following incubation, all soil samples (fumigated and non-fumigated) were extracted with 0.5M potassium sulfate (K_2_SO_4_) solution at a soil-to-solution ratio of 1:10. The mixtures were shaken for 30 min, centrifuged at 2000 rpm for 3 min, and filtered through a 0.45 μm membrane. The filtrate was diluted 10-fold and analyzed using a TOC analyzer. MBC was calculated as the difference in soluble organic carbon content between the fumigated and non-fumigated samples, divided by a conversion factor of 0.45.

NH_4_^+^-N and NO_3_^-^-N contents were determined using a flow analyzer (SEAL AutoAnalyzer 3, UK) following this procedure: 1) a 2 g soil sample wea weighted; 2) the sample was extracted with 2M potassium chloride (KCl) solution at a 1:5 soil-to-water ratio, and the dry soil weight for caculation was derived from the soil moisture content; 3) the mixture was shaken for 30 mmin, centrifuged at 2000 rpm for 3 min, and filtered; 4) the filtrate was analyzed directly.

**Table 1** The amendments of urea, pig manure, and rice straw of each treatment.

| Treatment | Urea | Pig manure | Rice straw |
| --- | --- | --- | --- |
|  | mg N g^-1^ dry soil | mg N g^-1^ dry soil | mg N g^-1^ dry soil |
| CK | 0 | 0 | 0 |
| CN | 0.08^a^ | 0 | 0 |
| 25%PN | 0.06 | 1.04 | 0 |
| 25%RN | 0.06 | 0 | 2.08 |
| 50%PN | 0.04 | 2.08 | 0 |
| 50%RN | 0.04 | 0 | 4.15 |

Note: ^a^The amendments were calculated based on a density of 1.20 g cm^-3^ and an effective soil layer depth of 0.20 m (equivalent to applying 200 kg N hm^-2^ in the field). The pig manure and rice straw particles contained a N content of 2% and 1%, respectively.

**Table S2** List of primers, sequence, and program processes

| Target gene | Primer and sequence | Program processes | Reference |
| --- | --- | --- | --- |
| *nirS* | cd3aF  GTSAACGTSAAGGARACSGG  R3cd  GASTTCGGRTGSGTCTTGA | 95℃ for 1 min × 1 cycle; 95℃ for 45 s, 55℃ for 45 s, 72℃ for 45 s × 40 cycles; | Throbck et al. (2004) |
| *nirK* | FlaCu  ATCATGGTSCTGCCGCG  R3Cu  GCCTCGATCAGRTTGTGGTT | 95℃ for 1 min × 1 cycle; 95℃ for 20 s, 63℃ for 30 s, 72℃ for 30 s × 40 cycles; | Throbck et al. (2004) |
| *nosZ* | F  CGYTGTTCMTCGACAGCCAG  1622R  CGSACCTTSTTGCCSTYGCG | 95℃ for 1 min × 1 cycle; 95℃ for 20 s, 58℃ for 30 s, 72℃ for 30 s × 40 cycles; | Throbck et al. (2004) |


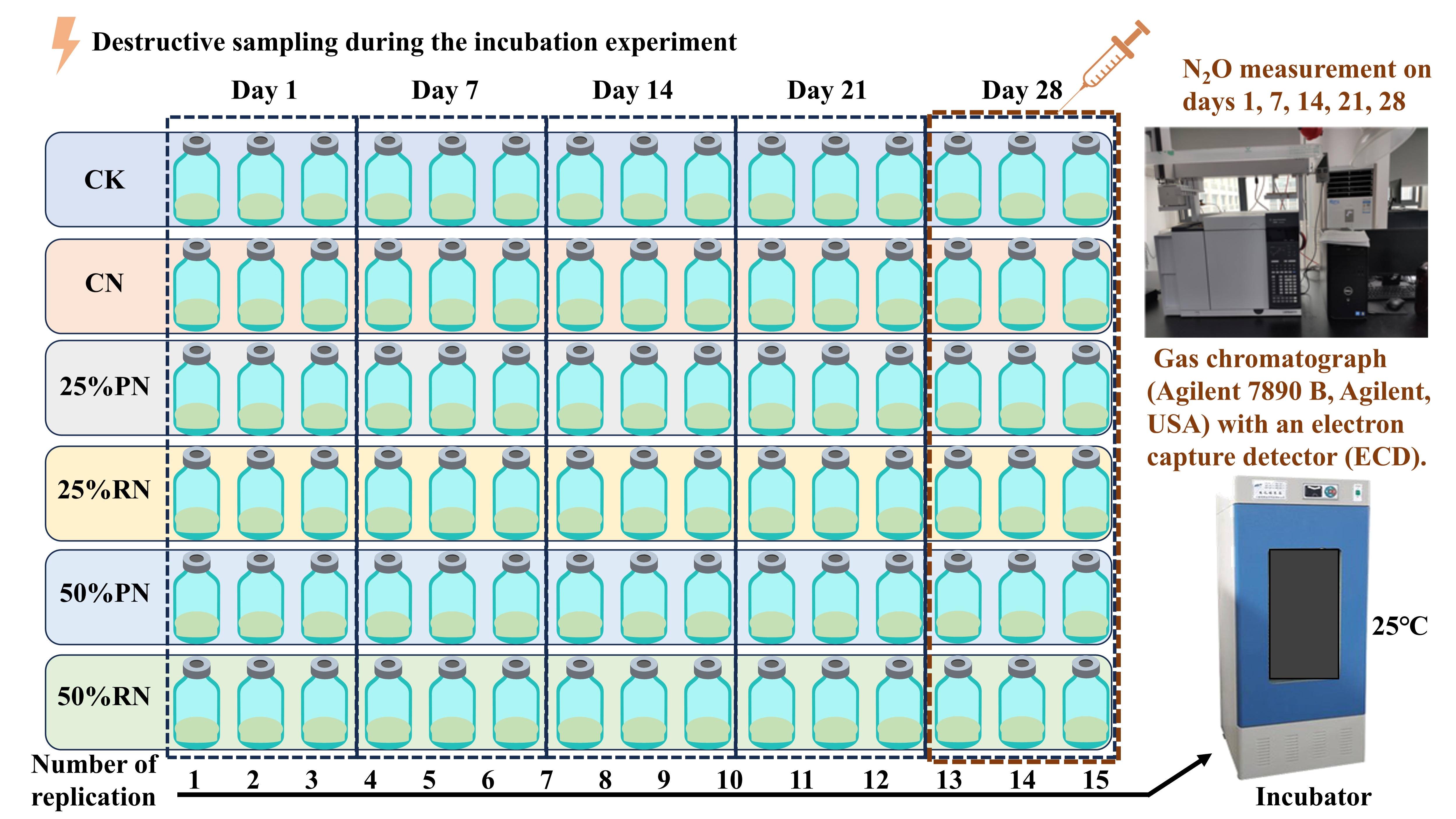


**FIGURE S1** The procedure of this incubation experiment. CK, no N fertilizer amendent; CN, amendment with urea only; 25%PN, 25% N derived from pig manure and 75% from urea; 25%RN, 25% N derived from rice straw and 75% from urea; 50%PN, 50% N derived from pig manure and 50% from urea; 50%RN, 50% N derived from rice straw and 50% from urea.


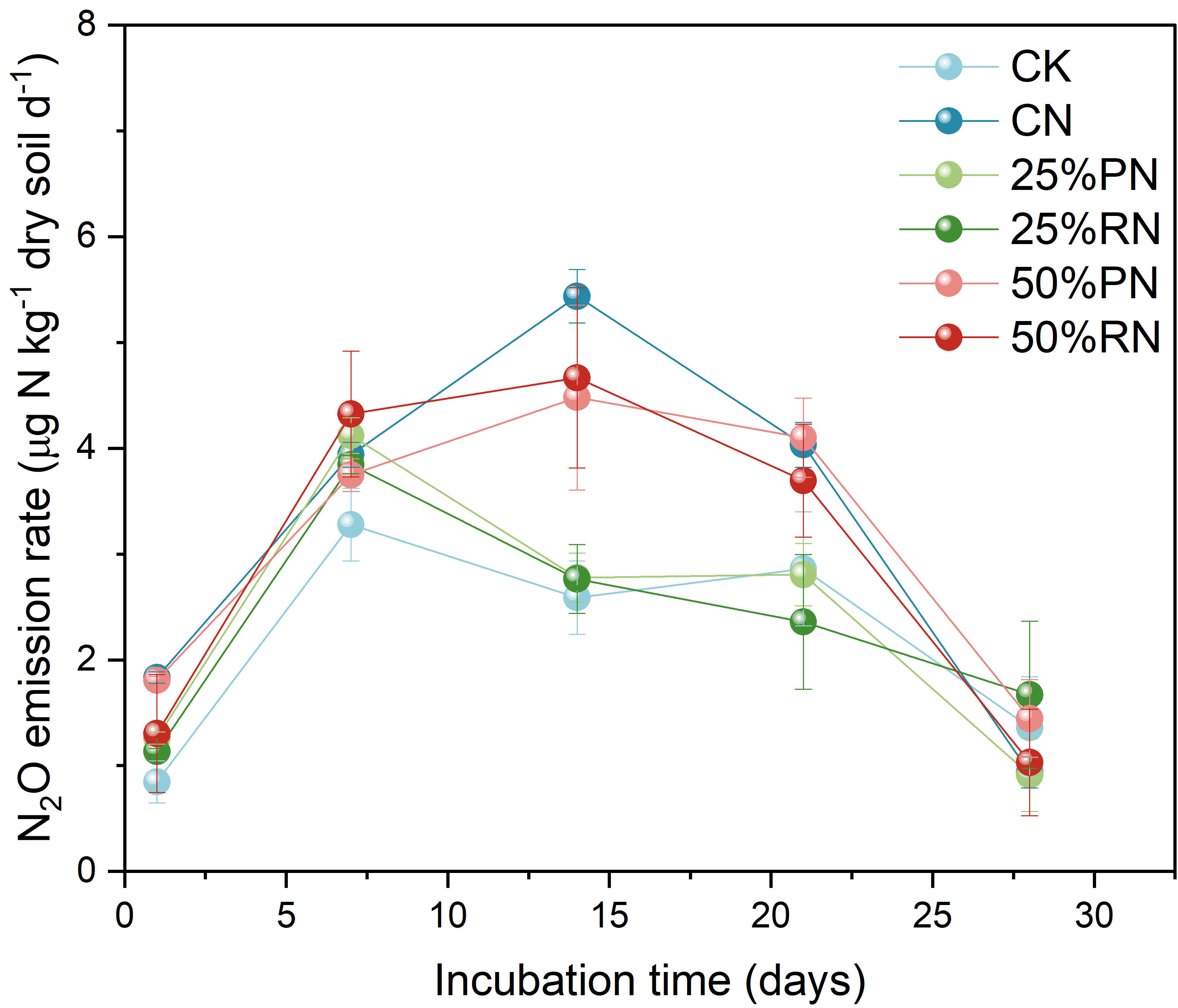


**FIGURE S2** N_2_O emission rate in tea plantation soils during incubation time. CK, no N fertilizer amendent; CN, amendment with urea only; 25%PN, 25% N derived from pig manure and 75% from urea; 25%RN, 25% N derived from rice straw and 75% from urea; 50%PN, 50% N derived from pig manure and 50% from urea; 50%RN, 50% N derived from rice straw and 50% from urea.


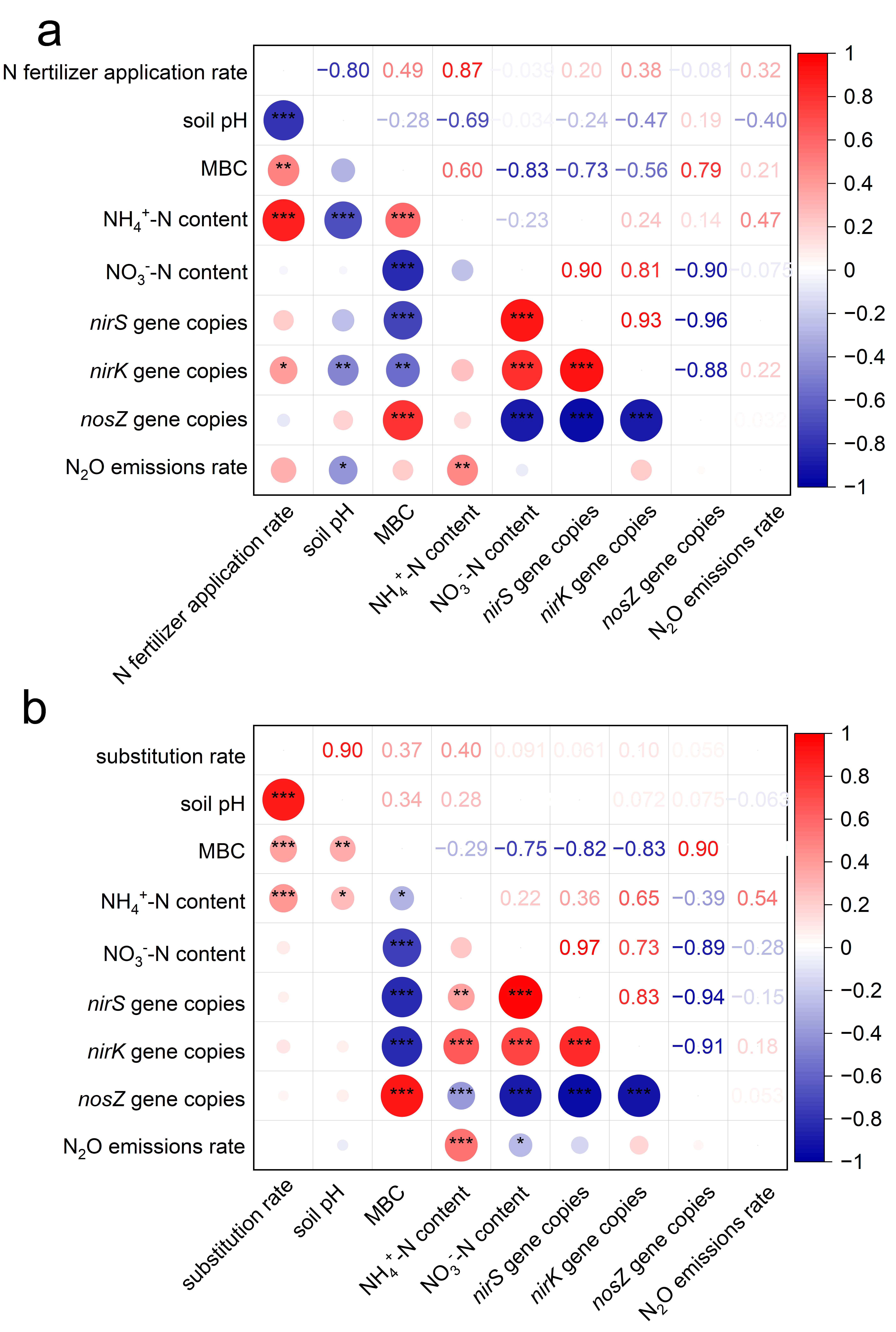


**FIGURE 3**

Relationships of fertilization and organic amendment with soil properties, denitrifying microbial genes (*nirS*, *nirK*, and *nosZ*), and N_2_O emission rates. The significance level was set at *, *p* < 0.05; **, *p* < 0.01; ***, *p* < 0.001, respectively.

**Reference**

Throbck, I.N., Enwall, K., Jarvis, A., Hallin, S. 2004. Reassessing PCR Primers Targeting nirS, nirK and nosZ Genes for Community Surveys of Denitrifying Bacteria with DGGE. *FEMS Microbiology Ecology*, **49**(3), 401-417.
